# A Combination of Conventional Transconjunctival Lower Blepharoplasty and Percutaneous Nanofat Grafting in a Young Chinese Population With Eyelid Bags and Tear Trough Deformities

**DOI:** 10.1111/jocd.70009

**Published:** 2025-01-23

**Authors:** Chuanbo Liu, Zhiyuan Wu, Liang Tang, Zijing Zhang, Jinsheng Li, Xiaofang Sun

**Affiliations:** ^1^ Department of Plastic and Cosmetic Surgery, Affiliated Hangzhou First People's Hospital Westlake University School of Medicine Hangzhou Zhejiang People's Republic of China; ^2^ Department of Plastic Surgery Wenzhou Ouhai District People's Hospital Wenzhou Zhejiang People's Republic of China; ^3^ The Fourth School of Clinical Medicine Zhejiang Chinese Medical University Hangzhou Zhejiang People's Republic of China; ^4^ Department of Dermatology The Second Affiliated Hospital of Jiaxing University Jiaxing Zhejiang People's Republic of China

**Keywords:** orbital fat removal, percutaneous nanofat grafting, retrospective study, transconjunctival lower blepharoplasty

## Abstract

**Objective:**

A simple and minimally invasive combined procedure, including transconjunctival orbital fat removal and transcutaneous resected orbital fat injection, was performed based on the anatomical characteristics of the lower eyelids in our young Chinese patients. Our study aimed to investigate the efficacy and safety of this procedure in our study population.

**Methods:**

In our retrospective study, a total of 183 consecutive patients underwent a combination of traditional transconjunctival blepharoplasty and nanofat grafting between February 2020 and June 2024. After transconjunctival septal fat removal, the resected orbital fat was processed into fat emulsion (nanofat) and then injected percutaneously into the depressed tear trough zone.

**Results:**

The mean age of the 183 patients was 26.9 years old, and the average surgery time was 36.1 min. The mean volume of nanofat injected for bilateral lower eyelids in each patient was 1.1 mL. All the patients were followed for at least 6 months after surgery, and the subjective patient satisfaction was 96.6% (175/183) with fewer complications. In the objective assessment of the surgical outcomes according to the five anatomical elements of lower eyelids, orbital fat prolapse, tear trough depression and dark circles were all improved, orbicularis prominence was more evident, while skin elasticity did not vary significantly after operation.

**Conclusion:**

Our study provided a simple, effective, and safe combination of transconjunctival lower blepharoplasty and percutaneous nanofat grafting for the treatment of lower eyelid bags with high patient satisfaction, quick recovery and low complication rate. Thus, this combined application may be a promising alternative choice for the young Chinese with lower eyelid bags.

## 1 Introduction

Patients seeking lower blepharoplasty often attribute their problem to a baggy appearance of the lower eyelids. With the development of the facial rejuvenation surgery, a better understanding of the anatomical structures of the periorbital region, including the lower eyelid, has been achieved [1]. Lower eyelid bags are a kind of complex and comprehensive problem caused by several anatomical changes [2, 3]. Apart from orbital fat herniation [2], the tear trough depression [2, 4, 5] is frequently present in the medial third part of the infraorbital rim. Other anatomical abnormalities such as dark circles [6], skin laxity [2] and fine wrinkles are also associated with eyelid bags. All these anatomical elements, both congenital and acquired with age, lead to a tired and aged appearance.

In order to restore a youthful contour, the lower blepharoplasty has evolved from classic fat excision to current fat preservation [3]. Fat repositioning and fat grafting are two major categories of surgical procedures [7, 8, 9, 10, 11] widely used to eliminate the protruding baggy appearance and to add tissue volume to improve the hollow infraorbital area, including the tear trough. Although both procedures can provide satisfactory cosmetic results, each has its own pros and cons. Compared with fat repositioning, autologous fat grafting following orbital fat removal in lower blepharoplasty has some merits [8, 12, 13, 14, 15, 16] including convenient operation, minimal invasiveness and quick recovery, but it may still carry risks [17] such as uneven skin contour in periorbital and perioral areas.

Many plastic surgeons have developed and streamlined individualized surgical methods to address the specific anatomical problems of eyelids confirmed in their population [12, 13, 14, 15, 16, 18, 19, 20]. Due to the anatomical characteristics of the lower eyelids in our young population, a combined application of two surgeries, transconjunctival orbital fat excision followed by autologous orbital fat transplantation, was adopted in our study. During fat grafting, the harvested orbital fat is processed into nanofat [14, 17, 21], which is more suitable for injection into the periorbital area. Our study aimed to validate the efficacy of this simple and minimally invasive combination of transconjunctival lower blepharoplasty and percutaneous nanofat injection in our young Chinese population.

## 2 Patients and Methods

The retrospective study of consecutive young patients for the treatment of lower eyelid bags was conducted between February 2020 and June 2024 at the Affiliated Hangzhou First People's Hospital, Westlake University School of Medicine. A total of 183 patients who had lower eyelid bags without skin laxity or with slight skin laxity were recruited for our study. All patients wanted to reduce their tired appearance and all were under the age of 40. Lower eyelids with excessive skin redundancy, a history of surgery or trauma to the infraorbital region within 1 year, the injection of filler within the previous 6 months, and other systemic diseases such as coagulopathy were the exclusion criteria. All the patients signed an informed consent and underwent a combined procedure including transconjunctival septal fat resection and transcutaneous resected fat grafting in our hospital. This study was approved by the Ethics Committee of our hospital and adhered to the tenets of the Declaration of Helsinki.

### 2.1 Patients' Information Collection

Patient demographics including age, gender, race was collected and standard preoperative photographs were taken. The operative time and injection volume of autologous resected septal fat were recorded immediately after surgery. The surgical history of a lower blepharoplasty (more than 1 year prior) was also obtained. All patients were followed up for at least 6 months, follow‐up period was calculated, postoperative photographs were taken and complications related to both septal fat removal and autologous fat transplantation were documented.

Patient satisfaction was subjectively rated by themselves on a 4‐point grade (worse being 1; low satisfaction or not satisfied being 2; satisfied being 3; very satisfied being 4) according to the therapeutic outcome at the end of follow‐up.

All the patients came to our clinic to address the problem of the lower eyelids, with the main complaint being the appearance of fatigue and aging. However, in addition to orbital fat herniation, tear trough deformity and dark circles were also common in most of the young Chinese patients. In order to objectively evaluate the improvement in aesthetic appearance before and after surgery, our study adopted five major components that contribute to the anatomy of the baggy eyelids in the Chinese population, referring to the scoring system of Goldberg et al. [2], Sadick et al. [4], and Cheng et al. [22]. Thus, orbital fat prolapse, tear trough depression, dark circles, orbicularis prominence, and loss of skin elasticity were analyzed in detail by blindly comparing the pre‐ and post‐operative photographs by the two independent observers. In addition, both sides of the lower eyelids should be assessed respectively, as the two sides of lower eyelid bags are usually asymmetrical in the same person. Each indicator of the five elements was graded into five scales, recording no involvement, mild, moderate, marked or severe. Therefore, the scale for each category was scored before and after surgery for each eyelid of all patients in this study. The number and percentage of eyelids with grades from no involvement to severe were then calculated.

### 2.2 Preoperative Design

Lower blepharoplasty is routinely performed with the patient in the supine position, which hides the three bulging septal fat pads. Therefore, the preoperative evaluation and planning of tranconjunctival lower blepharoplasty in our study was conducted with the patient in an upright position.

**FIGURE 1:**
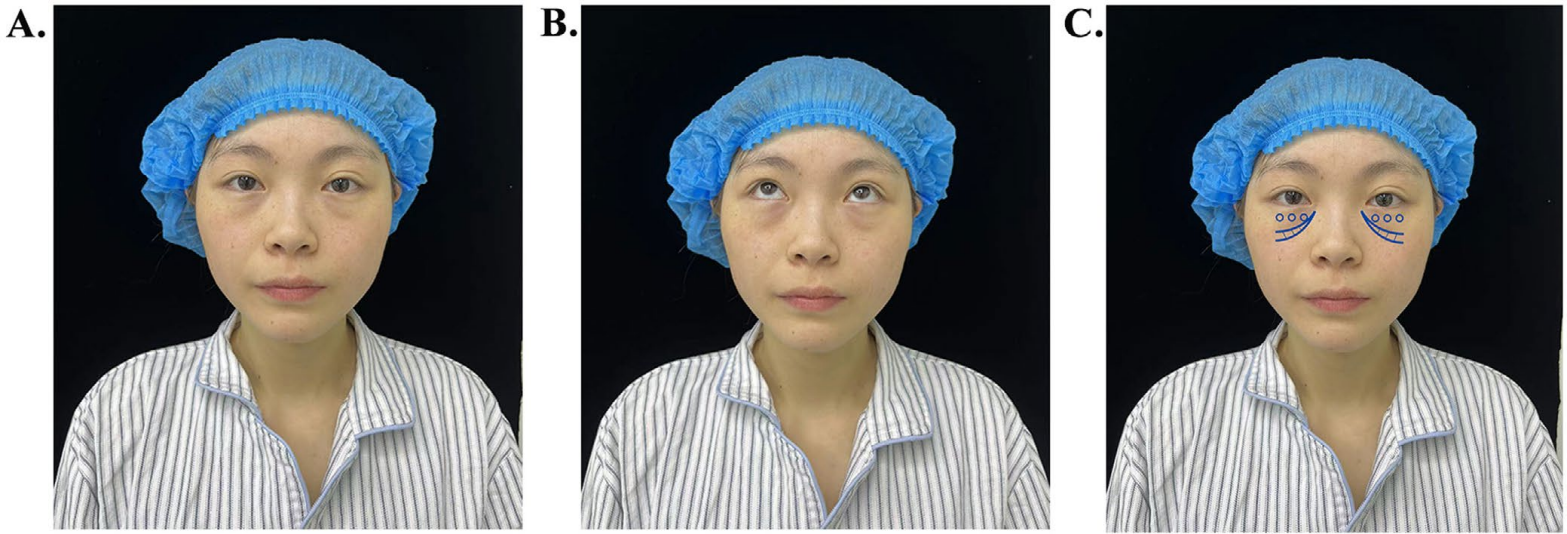
Preoperative design. In the upright position, the patients looked straight ahead (A) and look upwards (B), and the location of the orbital fat pads and the tear trough depression were marked (C).

In the sitting position, the patients were asked to look straight ahead and look upwards, then the location of the orbital fat was briefly marked for precise removal of redundant medial, central, and lateral fat pads. The zone of the tear trough depression was depicted in the sunken area of the anterior maxilla below the medial infraorbital rim and was the recipient area for autologous septal fat injection (Figure 1).

### 2.3 A Combined Technique of Transconjunctival Blepharoplasty and Nanofat Grafting

#### 2.3.1 Transconjunctival Resection of Orbital Fat Pads

**FIGURE 2:**
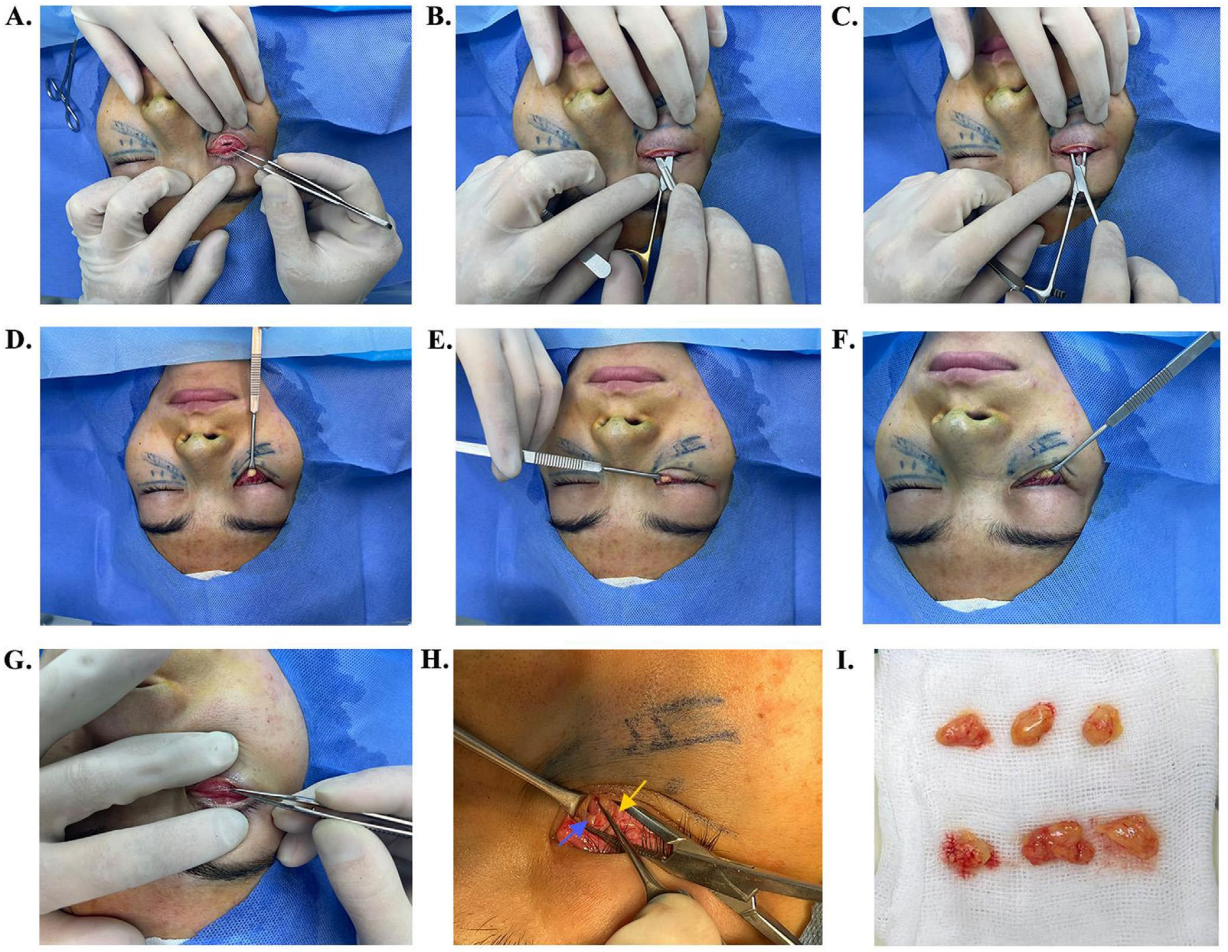
Transconjunctival resection of orbital fat pads. (A)Incision of the conjunctiva and capsulopalpebral fascia. (B, C) Dissection of the retro‐orbicularis oculi muscle using ophthalmic scissors and mosquito clamps. (D–F) Resection of the central, medial and lateral orbital fat pads. (G) Repositioning of the divided conjunctiva and capsulopalpebral fascia. (H) Avoiding damage to the inferior oblique muscle, as indicated by the forceps, during excision of the medial fat pad. The medial and central fat pads are marked with blue and yellow arrows respectively. (I) Placement of the three resected fat pads of each eyelid.

All of the surgeries in our study were carried out under local anesthesia, with the lower eyelid being retracted caudally by the assistant (Figure 2A). 2% lidocaine with 1:100000 epinephrine was injected into the subconjunctival layer, and then into each of the medial, central, and lateral orbital fat pads under the orbicularis oculi muscle and orbital septum. After 5 min of compression on the anesthetized area, the conjunctiva and capsulopalpebral fascia were both incised at the site of five millimeters inferior to the lower eyelid margin using a No. 11 blade (Figure 2A). The eyelid margin of the split conjunctiva was pulled back in a caudal direction by the assistant using a lid retractor. Blunt dissection (Figure 2B,C) was performed towards the infraorbital rim below the orbicularis oculi muscle using ophthalmic scissors and mosquito clamps to secure the orbital septum intact. After completion of the retro‐orbicularis oculi muscle dissection, the three orbital fat compartments were exposed by incising the orbital septum with a small window. The three fat pads were herniated with gentle pressure on the globe, and then the central, medial and lateral orbital fat pads were resected in sequence (Figure 2D–F). The medial fat pad was carefully excised to avoid damaging the inferior oblique muscle (Figure 2H). The predicted amount of lower eyelid fat was removed conservatively with the patient in the supine position, and then the amount of fat removed was confirmed to be appropriate for the appearance of the infraorbital region when the patient was in the sitting position. All of the resected fat pads were immediately placed on the sterile saline‐soaked gauze until further processing (Figure 2I). The incised conjunctiva and capsulopalpebral fascia were restored to their original position and left in place without any sutures (Figure 2G).

#### 2.3.2 Transcutaneous Resected Fat Injection for Tear Trough Deformity

**FIGURE 3:**
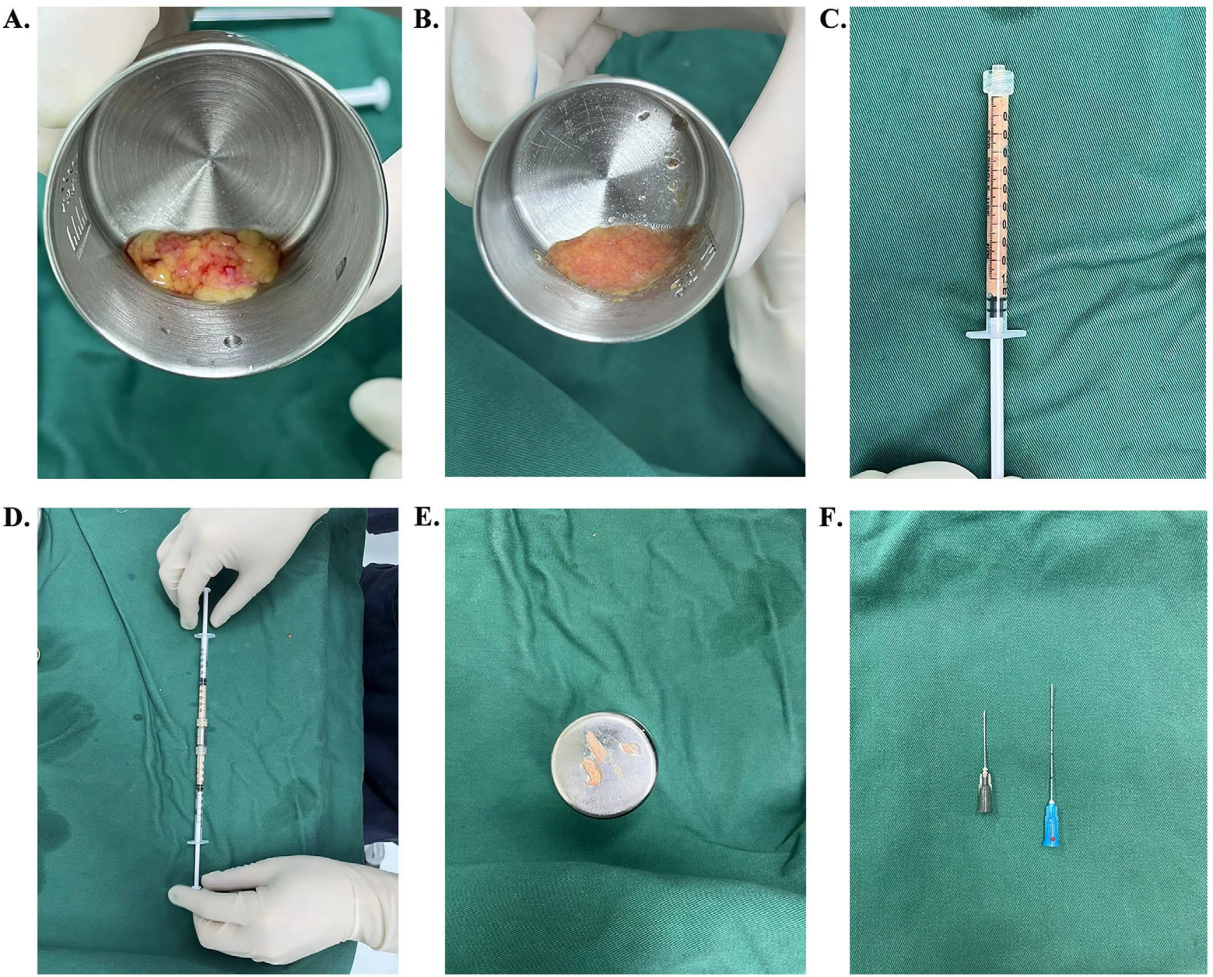
The emulsification process of the adipose tissue. (A, B) Chop the autologous orbital fat with scissors. (C) Transfer the minced fat into a 1 mL syringe. (D) Shift the fat between two syringes connected by a Luer‐Lock connector. (E) Remove the remaining the connective tissue that has blocked the Luer‐Lock connector. (F) 22 G sharp needle for skin puncture and 23 G blunt cannula for fat injection.

The resected orbital fat pads were washed with normal saline and chopped up with scissors (Figure 3A,B). Then the minced fat was transferred into two 1 mL syringes connected to each other by a Luer‐Lock connector with an internal diameter of 1.2 mm (Figure 3C). The fat was mechanically transferred at least 20 times between two syringes using a Luer‐Lock connector (Figure 3D). The yellow‐white fat emulsion obtained by shifting was nanofat. Nanofat [17, 21, 23], rich in adipose‐derived stem cells (ASCs), was injected to smooth the eyelid‐cheek junction and improve skin texture, resulting in a more even and natural contour of the infraorbital area. During this emulsification process of the adipose tissue, the remnants of the connective tissue blocking the Luer‐Lock connector were removed (Figure 3E).

**FIGURE 4:**
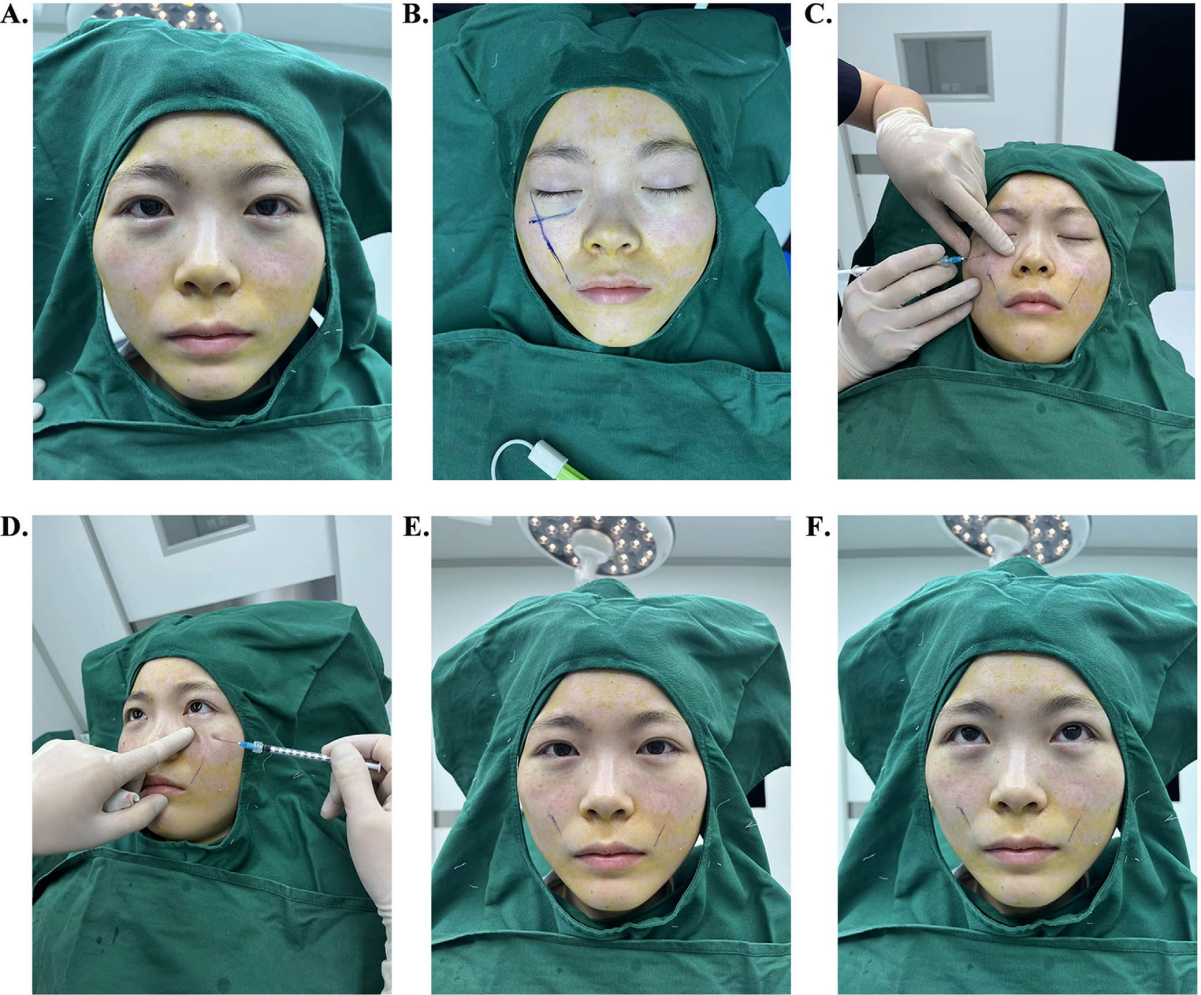
Transcutaneous resected fat injection for tear trough deformity. (A) The patient was in an upright position after completion of fat removal and processing. (B) The entry point for nanofat injection was marked. (C, D) Autologous fat was injected into the area of the tear trough in a fan shape using the surgeon's left index finger to perceive the injection layer and volume. (E, F) The desired aesthetic result was achieved after the combined procedure of lower blepharoplasty and nanofat grafting.

After completion of the transconjunctival removal of the orbital fat pads and processing of the fat emulsion, the patient adjusted his posture to a sitting position (Figure 4A). The percutaneous entry point for nanofat injection was marked at the intersection of the tear trough extension line and a line from the angulus oris to the lateral canthus (Figure 4B). This insertion point was then punctured with a 22 G sharp needle (as shown in Figure 3F) after local anesthesia with lidocaine. With the patient sitting upright, the nanofat injection was administered using a 23 G blunt injection cannula with a single side hole (as depicted in Figure 3F). During the procedure, the surgeon's left index finger was used to feel the injection layer of the needle tip and to sense the injection volume of autologous fat to avoid lump formation (Figure 4C,D). The nanofat graft was injected into different anatomical layers in multiple directions in the hollow area of the tear trough. In our study, the fat grafting was performed in three anatomical layers in a fan shape. The deep layer was located above the maxillary periosteum and approximately 25% of the total fat volume was transplanted into this layer. The middle layer was the space between the orbicularis oculi muscle and the periosteum, and about 50% of the total volume was injected into this layer. The superficial layer was a space between the skin dermis and the orbicularis oculi muscle, and a further 25% of the total fat was placed in this layer. The total volume of nanofat graft for both sides of the lower eyelids in one person was recorded. Once the fat injection was completed, digital pressure was slightly applied to shape the injection area until the desired aesthetic result was achieved when the patient looked forward and upward (Figure 4E,F).

### 2.4 Postoperative Management

A cold compress was applied for 20 min immediately after operation and used intermittently for the first 3 days to reduce postoperative bleeding and swelling. If necessary, eye drops were used to relieve postoperative discomfort. Oral antibiotics were not routinely administered after surgery. Patients were instructed not to put pressure on the surgical site for 1 week after surgery. Patients were instructed to minimize facial expressions, including smiling, and not to apply pressure to the surgical site for 1 week after surgery.

### 2.5 Statistical Analysis

Statistical analysis was performed with SPSS version 20 (IBM Corp, USA). Descriptions of quantitative variables were presented as mean with standard deviation, while qualitative variables were expressed as number and percentage. Five elements of each eye including orbital fat prolapse, tear trough depression, dark circles, orbicularis prominence and loss of skin elasticity were evaluated. And the scale of each indicator of the eyes before and after operation was documented. The number and percentage of eyelids with scales being no involvement, mild, moderate, marked or severe were calculated, and then the chi‐square test was analyzed for the qualitative variables to compare the differences of each index before and after surgery, The level of *p* < 0.05 was regarded as statistically significant.

## 3 Results

From February 2020 to June 2024, a total of 183 consecutive patients with 366 lower eyelid bags were enrolled in our study. All the patients received this simplified application of conventional transconjunctival blepharoplasty combined with resected nanofat grafting at the Affiliated Hangzhou First People's Hospital, Westlake University School of Medicine, and all the surgeries were performed by the attending surgeon, Chuanbo Liu.

### 3.1 General Information of the Study Population

The mean age of the 183 patients was 26.9 years old, and 173 (95%) were female. 180 (98%) of the patients were of Han nationality and only three (2%) were from other ethnic minorities. Of the 183 patients, four (2%) had a previous transconjunctival lower blepharoplasty several years ago and underwent their second surgery for recurrent lower eyelid bags.

All of the 183 patients were followed for at least 6 months after surgery based on the protocol, and the mean duration of follow‐up was 29.8 weeks. The average surgery time for the entire procedure including transconjunctival septal fat removal and percutaneous incisional fat injection was 36.1 min. The mean volume of total nanofat injected for bilateral lower eyelids in each patient was 1.1 mL.

These baseline demographic characteristics and surgery‐related data of our study population were listed in Table 1.

**TABLE 1:** General information of the 183 patients.

Variables	Value
Number of patients	183 (100%)
Age (years)	26.9 ± 3.9
Gender	
Male	10 (5%)
Female	173 (95%)
Race	
Han nationality	180 (98%)
Other ethnic minorities	3 (2%)
Surgical history of lower blepharoplasty (> 1 year ago)	
Yes	4 (2%)
No	179 (98%)
Follow‐up duration (weeks)	29.8 ± 3.8
Surgery time for the entire procedure (minutes)	36.1 ± 6.7
Injection volume of total fat for bilateral lower eyelids (mL)	1.1 ± 0.3

### 3.2 Assessment of Surgical Outcomes by Pre‐ and Post‐Operative Comparison

**FIGURE 5:**
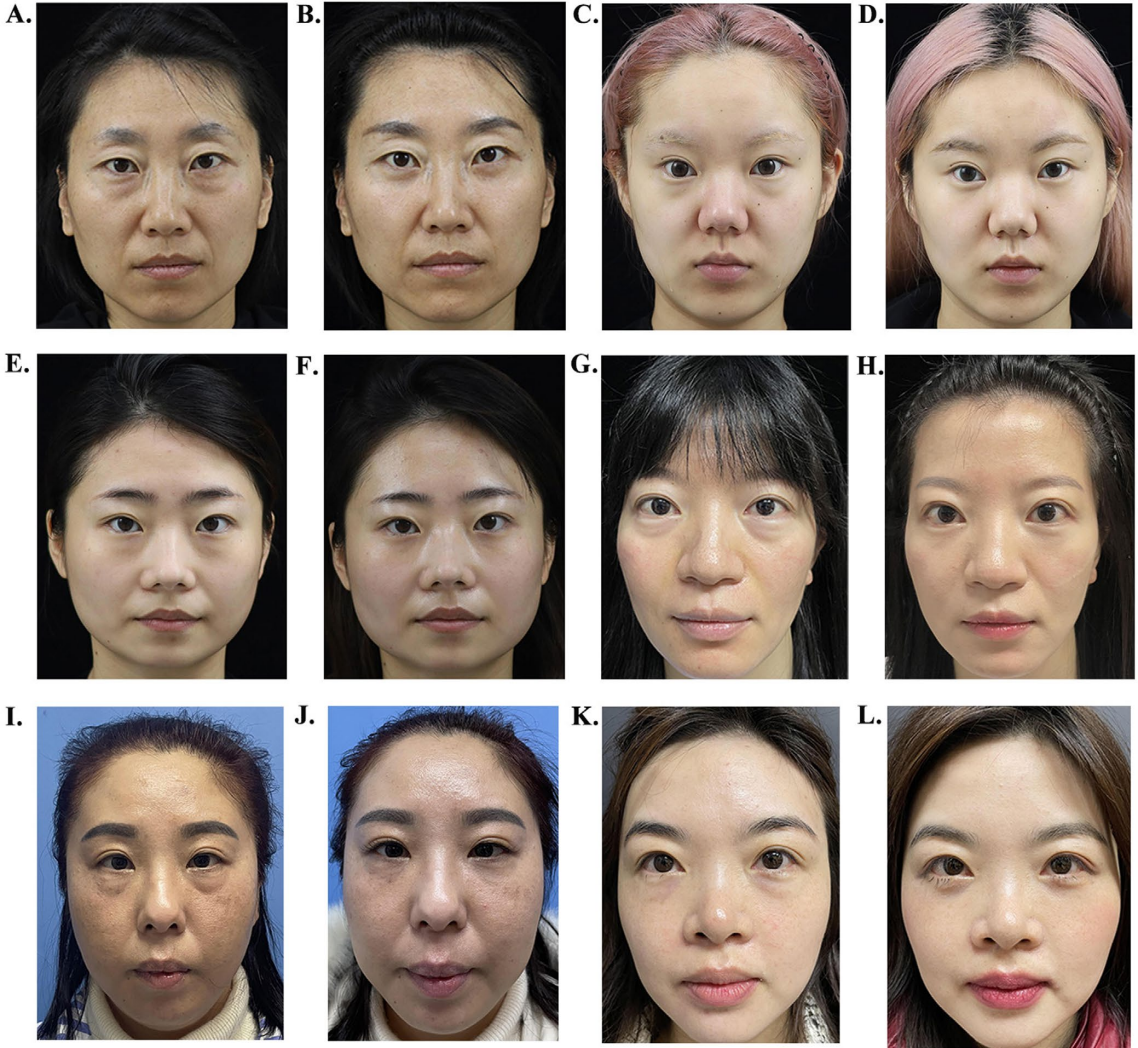
Preo‐ and post‐operative images of the patients. (A, B) Pre‐ and post‐surgery of a 38‐year‐old female; (C, D) Pre‐ and post‐surgery of a 21‐year‐old female; (E, F) Pre‐ and post‐surgery of a 26‐year‐old female; (G, H) Pre‐ and post‐surgery of a 36‐year‐old female; (I, J) Pre‐ and post‐surgery of a 40‐year‐old female; (K, L) Pre‐ and post‐surgery of a 35‐year‐old female.

The preoperative and postoperative images of the patients were shown in Figure 5.

Firstly, the subjective evaluation of the surgical outcomes was made in each patient based on patient satisfaction. Of the 183 patients, 175 (96.6%) were satisfied with the results of their surgery, with 75 (41.0%) being “very satisfied” and 100 (54.6%) “satisfied”. Only eight patients (4.4%) rated the outcomes of their operation as “low satisfaction or not satisfied”.

Secondly, the objective assessment of the operative results was conducted in each lower eyelid according to the scores of orbital fat prolapse, tear trough depression, dark circles, orbicularis prominence and loss of skin elasticity before and after surgery respectively. The number and percentage of lower eyelids with scales of no involvement, mild, moderate, marked or severe were recorded, and the differences of each index before and after operation were analyzed by chi‐square test as shown in Tables 2, 3, 4, 5, 6 below. These results of our study showed that the appearance of orbital fat prolapse (*p* < 0.01, Table 2), tear trough depression (*p* < 0.01, Table 3) and dark circles (*p* < 0.01, Table 4) were all improved after surgery. Postoperative orbicularis prominence was also more evident (*p* < 0.01, Table 5). However, there was no significant difference in loss of skin elasticity after operation (*p* > 0.05, Table 6), although someone felt more wrinkles in the lower lids.

**TABLE 2:** Preoperative and postoperative assessment of orbital fat prolapse in lower eyelids.

	No involvement no. (%)	Mild no. (%)	Moderate no. (%)	Marked no. (%)	Severe no. (%)	Total eyelids no. (%)
Preoperative assessment	0 (0%)	54 (14.8%)	189 (51.6%)	109 (29.8%)	14 (3.8%)	366 (100%)
Postoperative assessment	344 (94.0%)	22 (6.0%)	0 (0%)	0 (0%)	0 (0%)	366 (100%)

*Note:* Chi‐square test, *p* < 0.01.

**TABLE 3:** Preoperative and postoperative assessment of tear trough depression in lower eyelids.

	No involvement no. (%)	Mild no. (%)	Moderate no. (%)	Marked no. (%)	Severe no. (%)	Total eyelids no. (%)
Preoperative assessment	1 (0.3%)	47 (12.8%)	139 (38.0%)	155 (42.3%)	24 (6.6%)	366 (100%)
Postoperative assessment	115 (31.4%)	164 (44.8%)	81 (22.1%)	6 (1.6%)	0 (0%)	366 (100%)

*Note:* Chi‐square test, *p* < 0.01.

**TABLE 4:** Preoperative and postoperative assessment of dark circles in lower eyelids.

	No involvement no. (%)	Mild no. (%)	Moderate no. (%)	Marked n. (%)	Severe no. (%)	Total eyelids no. (%)
Preoperative assessment	34 (9.3%)	192 (52.5%)	134 (36.6%)	6 (1.6%)	0 (0%)	366 (100%)
Postoperative assessment	159 (43.4%)	158 (43.2%)	48 (13.1%)	1 (0.3%)	0 (0%)	366 (100%)

*Note:* Chi‐square test, *p* < 0.01.

**TABLE 5:** Preoperative and postoperative assessment of orbicularis prominence in lower eyelids.

	No involvement no. (%)	Mild no. (%)	Moderate no. (%)	Marked no. (%)	Severe no. (%)	Total eyelids no. (%)
Preoperative assessment	106 (29.0%)	203 (55.5%)	55 (15.0%)	2 (0.5%)	0 (0%)	366 (100%)
Postoperative assessment	28 (7.7%)	162 (44.3%)	166 (45.4%)	10 (2.7%)	0 (0%)	366 (100%)

*Note:* Chi‐square test, *p* < 0.01.

**TABLE 6:** Preoperative and postoperative assessment of loss of skin elasticity in lower eyelids.

	No involvement no. (%)	Mild no. (%)	Moderate no. (%)	Marked no. (%)	Severe no. (%)	Total eyelids no. (%)
Preoperative assessment	252 (68.9%)	79 (21.6%)	35 (9.6%)	0 (%)	0 (%)	366 (100%)
Postoperative assessment	249 (68.0%)	74 (20.2%)	40 (10.9%)	3 (0.8%)	0 (%)	366 (100%)

*Note:* Chi‐square test, *p* > 0.05.

### 3.3 Postoperative Complications and Management

Postoperative complications occurred in both transconjunctival fat removal and transcutaneous fat grafting procedures and the detailed information was given in Table 7.

**TABLE 7:** Postoperative complications in our study.

Complications	Number and percentage of the patients
Transient bloodshot eyes	5 (2.7%)
Chemosis	3 (1.6%)
Dry eye symptoms	1 (0.5%)
Submuscular nodule	1 (0.5%)
Increased fine wrinkles	10 (5.5%)
Undercorrection of the tear trough depression	5 (2.7%)
Infraorbital hollowness	2 (1.1%)
Recurrence of lower eyelid bags	3 (1.6%)

None of the patients in our study developed serious complications associated with transconjunctival blepharoplasty including infection, hematoma, retrobulbar hemorrhage, lid retraction, ectropion, and scleral show. No severe lipoinjection‐related complications, such as fat embolism, were reported during the follow‐up period.

Almost all the patients experienced mild to moderate bruising, edema or swelling of the periorbital area, which resolved spontaneously after one to 2 weeks without further treatment. Five patients had transient bloodshot eyes, three patients experienced chemosis, and one patient showed dry eye symptoms. All of these minor complaints mentioned above were transient and resolved after one to 3 weeks with the application of ophthalmic drops or ointment. In addition, one patient had a palpable but nonvisible submuscular nodule that regressed spontaneously within 2 months. Ten patients developed more fine wrinkles on their lower eyelids after surgery, but this didn't affect their overall satisfaction. Five patients had undercorrection of the tear trough depression and two patients had infraorbital hollowness, while three patients complained of recurrent lower eyelid bags due to residual orbital fat prolapse. Surgical repair was performed by either additional fat transplantation or transconjunctival septal fat resection after at least 6 months.

## 4 Discussion

Removal of herniated orbital fat alone during a conventional blepharoplasty can only improve the protruding appearance of the lower eyelids. Tear trough deformities and dark circles associated with eyelid bags are not corrected because this technique doesn't improve the anatomical volume loss of the tear trough depression and skin texture [3]. As a result, the current mainstream of lower blepharoplasty has been refined into techniques of fat preservation with conservative resection of the orbital fat [5]. Many techniques [7, 8, 9, 10, 11, 12, 13, 14, 15, 16, 18, 19, 20] of lower blepharoplasty, categorized as fat repositioning or fat grafting, not only eliminate the prominent baggy appearance of the lower eyelid, but also increase the volume of the tear trough depression in the midface. Each of these methods has its own merits and demerits respectively. If the appropriate procedure is chosen based on the characteristics of the patient's lower eyelid, a satisfactory surgical result can be achieved [3].

The fat transposition technique [7, 11] during transcutaneous or transconjunctival lower blepahroplasty was performed by the tear trough ligament release and septal fat reset. Many plastic surgeons [10, 18, 19] have modified their individual fat repositioning techniques by releasing the arcus marginalis and then advancing and fixing the orbital fat pedicles either in the subperiosteal, supraosteal, submuscular or even supramuscular plane. Although widely and effectively used in lower blepahroplasty, fat redistribution still has some drawbacks [13, 15], including the possible disruption of the middle lamella caused by extensive dissection. In addition, the learning curve for surgeons is steep due to the relative complexity of the technique. For these above reasons, some experts attempted to combine traditional lower blepharoplasty in conjunction with a minimally invasive fat grafting technique [12, 13, 14, 15, 16, 20, 24]. In this study, we provided a combined application of conventional transconjunctival lower blepharoplasty and transcutaneous resected orbital fat injection to address the concerns of lower eyelid bags in the young Chinese patients, which proved to be simple, effective and safe.

The main benefit of our combined application of traditional lower blepharoplasty and nanofat grafting was simplification, and the average total operative time was 36.1 min. This procedure met the desire of young patients for a minimally invasive operation with rapid recovery. Transconjunctival orbital fat removal and transcutaneous resected fat grafting were two separate but sequential steps in our technique. Both procedures were simple and less invasive, so the combined therapy was more acceptable to patients and the learning curve for surgeons was relatively short. The first step was to expose and incise the three orbital fat pads of each eyelid after completing the retro‐obicularis oculi muscle dissection. In this procedure, we didn't carry out an extensive dissection up to the orbital rim or a complete release of the tear trough ligament. All three fat compartments under the orbital septum were removed through one small incision. The second step was the process of fat emulsification [14, 17] and injection. The procedure was convenient because all the autologous fat used for grafting was harvested from the resected orbital fat only, with no further liposuction from other donor sites [8, 12, 14, 15, 20, 24]. After cutting the resected septal fat into pieces with scissors, the chopped fat was transferred into a 1 mL syringe. The fat emulsion was finally produced by mechanical transfer between two syringes using a Lock‐Leur connector. The single entry point for percutaneous fat injection was simply incised with an 22 G sharp needle with less injury, and then fat was injected in different layers of the tear trough groove in a fan shape using a 23 G blunt cannula.

In our study, following transconjunctival orbital fat removal, the resected fat was processed into nanofat and injected. The application of nanofat was one of the features of our combined surgery, as other research used various forms of autologous fat grafting, including free excised 2‐3 mm or even larger orbital fat transplantation [9, 11], fractionated fat or microfat injection [14, 16], or stromal vascular fraction gel injection [8]. In our study, patients ranged in age from 18 to 40 years, with an average age of 26.9 years, and 95% were female. There was no severe skin laxity or obvious wrinkles in the eyelids, and orbital fat herniation, tear trough deformity and dark circles were common in our young Chinese patients. Tear trough deformities in the young are usually associated with congenital maxillary retrusion in addition to the cutaneous, muscular and ligamentous structures of the infraorbital region [4, 5] while dark circles [6] are usually associated with the thin and transparent skin of the lower eyelids. Nanofat is rich in adipose‐derived stem cells (ASCs) with a good capacity for proliferation and differentiation [17, 21, 23], and many studies [8, 14, 17] have confirmed the effect of nanofat on skin rejuvenation. By combining with traditional lower blepharoplasty, percutaneous nanofat injection was confirmed to be effective in our study, with overall subjective patient satisfaction as high as 96.6%. Tear trough deformities were improved (*p* < 0.01, Table 3), while dark circles were significantly alleviated (*p* < 0.01, Table 4) after a few months of nanofat filling in our young population with lower eyelid bags.

These favorable results may be related to the measurements we took during the nanofat grafting in our study. Nanofat [17, 21] was a yellow‐white fat emulsion harvested after mechanical mincing and transfer of the resected septal fat. Nanofat has lost its normal adipose tissue structure and the main concern with nanofat grafting is the unpredictable rate of fat retention [13, 23]. Therefore, the volumetric capacity of the nanofat is considered to be limited [17]. However, in our study, although the exact figure was not calculated, the fat retention rate might still be acceptable. To improve the effect of the operation, nanofat injection was distributed in multiple anatomical layers and in small amounts increased the blood supply to the transplanted fat [15]. In addition, the tear trough ligament was released to some extent during the process of multilayer injection using a blunt cannula [20]. Thus, the increased volume of fat in the subperiosteal, submuscular and subcutaneous layers provided the supporting effect [15]. Furthermore, the lower eyelid was less involved in facial expression movements [12] and patients were instructed to minimize facial expressions, especially smiling, for 1 week after surgery. These three factors, taken together, may have favored the volume retention rate of the nanofat graft [12, 23]. However, further research is needed to clarify the complex causes of this speculation. Because a small amount of nanofat was injected into the superficial subcutaneous layer, dark circles were also reduced, similar to the results of other studies [12, 15]. By communicating with patients during follow‐up visits, another underlying cause of high patient satisfaction may exist. The main purpose of fat grafting after orbital fat removal was to mold a youthful contour with a smooth lid‐cheek junction rather than simply to augment the volume. Hence, the flat appearance of the lower eyelids without the convex‐concave deformity (protruding fat and tear trough groove) increased patient satisfaction, even though the infraorbital zone was slightly hollow.

Based on the anatomical characteristics of the eyelid bags in our study population, a five‐factor evaluation, modified from the scoring system of Goldberg et al. [2], Sadick et al. [4], and Cheng et al. [22], was adopted to more objectively and comprehensively assess our surgical outcomes. Apart from orbital fat prolapse, tear trough depression and dark circles, the objective assessment of orbicularis prominence and skin elasticity before and after surgery was also important and had an impact on patient satisfaction. The orbicularis prominence, also known as pretarsal roll, is considered a significant symbol of youth in the Asian population [25]. After removal of orbital fat, a smoother lower eyelid probably made the orbicularis muscle more prominent. Our study confirmed that the prominence of the orbicularis was enhanced after surgery (*p* < 0.01, Table 5), which is consistent with another similar study [13]. Although 10 patients developed more fine wrinkles in their lower eyelids after surgery, there was still no significant difference in the loss of skin elasticity after operation (*p* > 0.05, Table 6). However, this didn't affect their overall satisfaction. On the one hand, this was related to the choice of surgical technique, and patients were informed of the advantages and disadvantages of transconjunctival and transcutaneous approaches to lower blepharoplasty respectively. It was therefore understandable that the limitations of transconjunctival blepharoplasty could lead to increased wrinkling after surgery [13]. On the other hand, nanofat injection could improve skin texture through the rejuvenating effect of ASCs [14, 17, 23]. This could compensate for the increased skin relaxation caused by the transconjunctival approach.

This study confirmed that our combination application of lower blepharoplasty and nanofat grafting is safe with few complications. No major complications such as infections or vascular thrombosis occurred in our population. In our study, the incidence of minor complications was low as shown in Table 7, and only one patient developed a liponodule. It was not visible but palpable and resolved with any therapy. In order to prevent and reduce the complications associated with fat embolism [12, 15], a very tiny volume dose of fat was delivered with each syringe push. What is more, the injection area was gently massaged during the fat grafting process. Besides, in order to place the autologous fat in the precise location to correct the tear trough deformity, the nanofat injection was performed with the patient in an upright position [16]. Due to the different position and volume of the tear trough in the supine and sitting positions, this maneuver was superior to lipoinjection through the same incision [12].

There are still some limitations in our study. Firstly, it was a retrospective study and there were some inherent flaws. The study protocol was confirmed post‐operatively, so pre‐ and post‐operative photographs were used to evaluate the aesthetic outcome of lower eyelid bags. It also failed to take photos of the patient making facial expressions, such as laughing, which were the best way to assess the safety of superficial injection [26]. In addition, some of the photos were taken with an iPhone against different backgrounds, and the lighting was not consistent before and after surgery, which could affect the assessment of surgical outcomes. Moreover, dark circles were evaluated and graded in five levels, from none to severe, and were not classified as hyperpigmented, vascular or mixed dark circles [6] according to the photographs. Therefore, prospective research with longer follow‐up may provide more persuasive evidence. Secondly, it was a non‐controlled study and we did not directly compare the outcomes and benefits of traditional transconjunctival lower blepharoplasty with fat repositioning versus fat grafting. Instead, Table 8 presents a comparative analysis of surgical techniques from various studies, encompassing surgical outcomes, patient satisfaction, and complications. Each method has its inherent advantages and disadvantages. Consequently, a case–control study should be conducted to offer a more definitive perspective on the comparative efficacy between fat repositioning and fat grafting. Thirdly, the retention rate of the grafted nanofat was not quantified, although patient satisfaction was high and the improvements in tear trough deformities were satisfactory. In further studies, the application of three‐dimensional assessment [27] can more precisely measure the infraorbital volume before and after lower blepharoplasty and then assist to objectively evaluate the fat retention rate.

**TABLE 8:** Comparison of some surgical techniques using traditional transconjunctival lower blepharoplasty with fat reposition or with autologous fat grafting.

Author and year of publication	Surgical technique	Number of patients and follow‐up duration	Assessment of surgical results	Patient satisfaction	Postoperative complications
Wong CH et al. [11] 2017	Release of the tear trough ligament via the transconjunctival approach and excised orbital fat fixation with percutaneous sutures	54 patients, 5 to 38 months	/	High satisfaction (no exact data available)	Revision (2%), corneal abrasion (2%), tiny bulge (2%), palpability/lumpiness of the grafted fat in the initial postoperative period (9%)
Kim HS et al. [13] 2019	Transconjunctival fat removal followed by resected fat grafting	229 patients, 1 to 80 months	Goldberg scores for orbital fat prolapse, tear trough depression, skin transparency, and triangular malar mound improved. The orbicularis prominence worsened. Skin elasticity deteriorated	97.8% were satisfied	Deep wrinkles (7.4%), fine winkles (60.7%), transient darkening of the lower eyelids (37.6%), infraorbital hollowness (3.0%), supraorbital hollowness (0.4%), bloodshot eye (0.4%), intermittent orbicularis oculi muscle cramping (1.3%), infraorbital hardening of subcutaneous nodule lasting > 1 month (3.0%), conjunctival incision site granuloma (2.6%), burn (0.4%), transient ectropion (0.4%)
Jiang S et al. [8] 2020	Transconjunctival eye bag removal and injection of stromal vascular fraction gel	74 patients, 3–12 months	/	/	Revision (4%), tiny bulge (2 patients, 2.7%)
Goldberg RA [10] 2000	Transconjunctival orbital fat ransposition in a subperiosteal plane	24 patients, 6–30 months	No objective measurements	Excellent patient acceptance (no exact data available)	Various degrees of hardening of the fat pedicle in the first 3 months (no exact data available)
Majidian Ba M et al. [19] 2021	Transconjunctival fat transposition above the orbicularis muscle	41 patients, a minimum of 44 months	Improvement in tear trough deformity, wrinkling and the contour of the lower eyelid	A high satisfaction (no exact data available)	Purpura and edema (no exact data available), transient blurring of vision (2%), bruising, swelling, and redness (few patients, no exact data available)
Gu J et al. [18] 2024	Internal fixation of redistributed orbital fat in transconjunctival lower blepharoplasty	22 patients, 10 to 14 months	Goldberg score for eyelid bags and tear trough deformity improved	85% with eyelid bags and 75% with TTD improved. The remaining patients were also satisfied	No hematoma, ectropion or midface numbness
Our study 2024	Traditional transconjunctival lower blepharoplasty and percutaneous nanofat grafting	183 patients, 6–10 months	Orbital fat prolapse, tear trough depression and dark circles improved. Orbicularis prominence was more evident. Skin elasticity did not vary	96.6% were satisfied	Transient bloodshot eyes (2.7%), chemosis (1.6%), dry eye symptoms (0.5%), submuscular nodule (0.5%), increased fine wrinkles (5.5%), undercorrection of the tear trough depression (2.7%), infraorbital hollowness (1.1%), recurrence (1.6%)

## 5 Conclusion

Our study offered a combination of conventional transconjunctival lower blepharoplasty and percutaneous excised orbital fat grafting to treat the lower eyelid bags and tear trough deformities based on the anatomical characteristics of our young Chinese population. This combined technique was simple and minimally invasive, and favorable results were achieved with high patient satisfaction, rapid recovery and low complication rate. Therefore, this combined application of traditional blepharoplasty and nanofat grafting may be a promising alternative surgical candidate for the young Chinese with lower eyelid bags to yield a satisfactory treatment outcome.

## Author Contributions

C.L., J.L., and X.S. designed the research study; C.L., Z.W., and L.T. performed the research; Z.Z. and J.L. analyzed the data; C.L. wrote the paper; J.L. and X.S. reviewed and edited the paper. All authors have read and approved the final manuscript.

## Ethics Statement

This retrospective study was approved by the Ethics Committee of Affiliated Hangzhou First People's Hospital, Westlake University School of Medicine (ZN‐20240401‐0109‐01) and adhered to the tenets of the Declaration of Helsinki.

## Conflicts of Interest

The authors declare no conflicts of interest.

## Data Availability

The data that support the findings of this study are available on request from the corresponding author. The data are not publicly available due to privacy or ethical restrictions.
